# A Rotating-Coil Magnetometer for Scanning Transversal Field Harmonics in Accelerator Magnets

**DOI:** 10.1038/s41598-018-37371-3

**Published:** 2019-02-06

**Authors:** Pasquale Arpaia, Gianni Caiafa, Stephan Russenschuck

**Affiliations:** 1 Instrumentation and Measurement for Particle Accelerators Laboratory, Department of Electrical Engineering and Information Technology, University of Napoli Federico II, Naples, Italy; 2 Magnetic Measurements Section, Technology Department, European Organization for Nuclear Research, Geneva, Switzerland

## Abstract

This paper presents a rotating-coil magnetometer that was designed and validated for scanning local transversal field harmonics, required for extracting so-called pseudo-multipoles in accelerator magnets. The magnetometer consists of four layers of flexible printed circuits with a track thickness of 40 μm. The design aimed at maximizing the sensitivity factors for field harmonics up to order 13 and at a compensation ratio for the main component in the same range of what is achievable with standard rotating coils. Key innovative features of the induction coil are the shape for minimizing the sensitivity to the longitudinal field component and the manufacturing technology. The design, the uncertainty analysis of the manufacturing tolerances, as well as preliminary application results are presented.

## Introduction

For magnetic measurements of accelerator magnets, the induction coil magnetometer is still the best transducer in terms of linearity, repeatability, reliability, and accuracy. Induction coils are applied to measure the field strength, direction, and field errors expressed as higher-order field harmonics^1,2^.

Magnetic measurements of accelerator magnets are usually performed with shafts containing a number of induction coils that are longer than the magnet length, and, therefore, cover also the fringe field regions. In fact, measuring the integrated transversal field components is often sufficient to validate the design and characterize the magnet, in particular for reproducibility in larger series. In other cases, the local field distribution measurement is required. This is the case for fringe-field dominated magnets and when the measurements are to be used for track reconstruction in spectrometers. Fringe field-dominated magnets are short magnets with relatively wide apertures, where the effect of the magnet ends is not negligible^3,4^. The knowledge of the local field distribution in the magnets is also important for the study of the beam dynamics of insertion regions where the *β*-function changes rapidly^5^. The field distributions at the magnet extremities cannot be developed into Fourier series (i.e. the classical field harmonics), because the trigonometric functions do not constitute a complete orthogonal function set of the field solution^6^. This gives rise to Fourier-Bessel series and the so-called pseudo-multipoles^7,8^, which depend on the magnetic field variation along the magnet axis. There are several established techniques for acquiring the local field distribution^9^. One possibility is to measure the longitudinal profile by mapping the magnet bore with a 3D Hall sensor^10,11^ mounted on a displacement stage or by using magneto-electric flux gate or absolute magnetometry^12,13^. Another solution is to use a translating-coil scanner on the magnet mid-plane^14^. In the latter case, however, the transversal resolution (and the highest order of the field harmonics) is limited by the track widths of the single coils. In^6^, it was proven that the classical rotating-coil magnetometers cannot be used in regions where a significant longitudinal field component is present. The extraction of pseudo-multipoles from transversal field measurements on a reference radius requires a coil that intercepts only the radial field component, and thus is free of the voltage induced by the longitudinal field component. The main objective for the coil design is to achieve the same resolution and measurement uncertainty of the standard rotating coils with a signal-to-noise ratio of about 60 dB^15^.

In this paper, we propose a new concept of a short, rotating-coil magnetometer that does not intercept the longitudinal field component. The coil is designed as a four-layer, flexible printed circuit, with 40 μm thick tracks and 50 μm electrical insulation between them. The mathematical formulation, the measurement principle, and the measurement method with its numerical validation are presented in the Section “Method”. In the Section “Results”, the sensor design, the computation of the coil-sensitivity factors, the uncertainty analysis of the main coil parameters, the sensor production, and the validation experiments are presented.

## Method

### Mathematical Formulation

The scaling laws derived from the integrated (2D) field harmonics in accelerator magnets cannot be used in the 3D case, because these field harmonics do not constitute a complete, orthogonal function set of the 3D Laplacian. The entire theory of 2D field harmonics, is based on a complex potential determined by the integral field quantities in the magnet. Applying the concept of pseudo-multipoles^4,6^, the field distribution in the end-regions of the magnet can be reconstituted from measurements on the boundary surface, i.e., the transversal multipole field errors measured by a relatively short, (short with respect to the magnet length and compared to the standard harmonic coils often covering the entire magnet and its fringe-field regions), saddle-shaped, induction coil. In a simply-connected, cylindrical domain, free of magnetized material and current sources, the field components can be calculated from a magnetic scalar potential *ϕ*_*m*_ obeying the Laplace equation

1 $${\nabla }^{2}{\varphi }_{m}=\frac{1}{r}\frac{\partial }{\partial r}(r\frac{\partial {\varphi }_{m}}{\partial r})+\frac{1}{{r}^{2}}\frac{{\partial }^{2}{\varphi }_{m}}{\partial {\phi }^{2}}+\frac{{\partial }^{2}{\varphi }_{m}}{\partial {z}^{2}},$$

where *r*, *φ* and *z* are the coordinates of a cylindrical reference system. Eigensolutions are given by a Fourier-Bessel series that can be approximated by the double sum

2 $${\varphi }_{m}(r,\phi ,z)=\sum _{k=0}^{\infty }\,\sum _{n=1}^{\infty }\,{r}^{n+2k}({C}_{n+2k,n}(z)\,\sin \,(n\varphi )+{D}_{n+2k,n}(z)\,\cos \,(n\varphi )),$$

where *C*_*n* + 2*k*,*n*_(*z*) and *D*_*n* + 2*k*,*n*_(*z*) are coefficients to be determined^16,17^. Inserting this expression into the Laplace equation yields a recursive equation for the coefficients. We obtain^6^

3 $${\varphi }_{m}(r,\phi ,z)=\sum _{n=1}^{\infty }\,{r}^{n}({\tilde{C}}_{n}(r,z)\,\sin \,(n\phi )+{\tilde{D}}_{n}(r,z)\,\cos \,(n\phi )),$$

where ${\tilde{C}}_{n}(r,z)$ and ${\tilde{D}}_{n}(r,z)$ are the normal and skew components given by

4 $$\begin{array}{rcl}{\tilde{C}}_{n}(r,z) & = & {C}_{n,n}(z)-\frac{{C}_{n,n}^{\mathrm{(2)}}(z)}{\mathrm{4(}n+\mathrm{1)}}{r}^{2}+\frac{{C}_{n,n}^{\mathrm{(4)}}(z)}{\mathrm{32(}n+\mathrm{1)(}n+\mathrm{2)}}{r}^{4}+\ldots ,\\ {\tilde{D}}_{n}(r,z) & = & {D}_{n,n}(z)-\frac{{D}_{n,n}^{\mathrm{(2)}}(z)}{\mathrm{4(}n+\mathrm{1)}}{r}^{2}+\frac{{D}_{n,n}^{\mathrm{(4)}}(z)}{\mathrm{32(}n+\mathrm{1)(}n+\mathrm{2)}}{r}^{4}+\ldots ,\end{array}$$

and where the superscript (m) denotes the *m*-derivative in the *z*-coordinate, ${C}_{n,n}^{(m)}(z):={\partial }^{m}{C}_{n,n}(z)/\partial {z}^{m}$. The field components within the bore of the magnet are given by

5 $${B}_{r}=-\,{\mu }_{0}\frac{\partial {\varphi }_{m}}{\partial r},\,{B}_{\phi }=-\,{\mu }_{0}\frac{1}{r}\frac{\partial {\varphi }_{m}}{\partial \phi },\,{B}_{z}=-\,{\mu }_{0}\frac{\partial {\varphi }_{m}}{\partial z},$$

where *μ*_0_ is the permeability of free space. Therefore

6 $${B}_{r}(r,\phi ,z)=-\,{\mu }_{0}\sum _{n=1}^{\infty }\,{r}^{n-1}({\bar{C}}_{n}(r,z)\,\sin \,(n\phi )+{\bar{D}}_{n}(r,z)\,\cos \,(n\phi )),$$

7 $${B}_{\phi }(r,\phi ,z)=-\,{\mu }_{0}\sum _{n=1}^{\infty }\,n{r}^{n-1}({\tilde{C}}_{n}(r,z)\,\cos \,(n\phi )+{\tilde{D}}_{n}(r,z)\,\sin \,(n\phi )),$$

8 $${B}_{z}(r,\phi ,z)=-\,{\mu }_{0}\sum _{n=1}^{\infty }\,{r}^{n-1}(\frac{\partial {\tilde{C}}_{n}(r,z)}{\partial z}\,\sin (n\phi )+\frac{\partial {\tilde{D}}_{n}(r,z)}{\partial z}\,\cos \,(n\phi )),$$

where

9 $${\bar{C}}_{n}(r,z)=n{C}_{n,n}(z)-\frac{(n+\mathrm{2)}{C}_{n,n}^{\mathrm{(2)}}(z)}{\mathrm{4(}n+\mathrm{1)}}{r}^{2}+\frac{(n+\mathrm{4)}{C}_{n,n}^{\mathrm{(4)}}(z)}{\mathrm{32(}n+\mathrm{1)(}n+\mathrm{2)}}{r}^{4}+\ldots \,.$$

Easier results are obtained when expressing the vertical field component on the horizontal plane:

10 $$\begin{array}{rcl}-\frac{1}{{\mu }_{0}}{B}_{\phi }(r,\phi =0,z) & \approx  & {C}_{1,1}(z)-\frac{{C}_{1,1}^{(2)}(z)}{8}{r}^{2}+\frac{{C}_{1,1}^{(4)}(z)}{192}{r}^{4}-\frac{{C}_{1,1}^{(6)}(z)}{9216}{r}^{6}+\frac{{C}_{1,1}^{(8)}(z)}{737280}{r}^{8}\\  &  & +\,3{C}_{3,3}(z){r}^{2}-\frac{3{C}_{3,3}^{(2)}(z)}{16}{r}^{4}+\frac{3{C}_{3,3}^{(4)}(z)}{640}{r}^{6}-\frac{3{C}_{3,3}^{(6)}(z)}{64512}{r}^{8}\\  &  & +\,5{C}_{5,5}(z){r}^{4}-\frac{5{C}_{5,5}^{(2)}(z)}{24}{r}^{6}+\frac{5{C}_{5,5}^{(4)}(z)}{1344}{r}^{8}-\ldots \\  &  & +\,7{C}_{7,7}(z){r}^{6}-\frac{7{C}_{7,7}^{(2)}(z)}{32}{r}^{8}-\ldots \\  &  & +\,9{C}_{9,9}(z){r}^{8}-\ldots \\  &  & \vdots \end{array}$$

This series is truncated at *n* = 9 because it already requires the 8-th derivative of the leading dipole term *C*_1,1_ with respect to the longitudinal coordinate *z*. It can be seen that the second derivative of the leading term, ${C}_{1,1}^{\mathrm{(2)}}$ gives rise to a *r*^2^ dependence of the field as the term with coefficient *C*_3,3_. In other words, the dipole field roll-off in the magnet extremities yields a radial field dependence that resembles a (pseudo) sextupole.

A way to extract the pseudo-multipoles from boundary data is to measure the function ${\tilde{B}}_{n}({r}_{0},z)$ that is a convolution of the transversal field harmonics *B*_*n*_(*r*_0_, *z*) with the kernel defined by the sensitivity of the induction coil, (for coil lengths converging to zero, this kernel would converge toward the Dirac delta distribution and a deconvolution of the measured signal would no longer be necessary), that is displaced step-by-step along the magnet axis, ${\tilde{B}}_{n}({r}_{0},z)={B}_{n}({r}_{0},z)\ast {k}_{n}({r}_{0},z)$, and then to solve

11 $$\begin{array}{rcl}{B}_{n}({r}_{0},z) & = & -{\mu }_{0}\,{r}_{0}^{n-1}\,{\bar{C}}_{n}({r}_{0},z)=-\,{\mu }_{0}\,{r}_{0}^{n-1}(n{C}_{n,n}(z)-\frac{(n+\mathrm{2)}{C}_{n,n}^{\mathrm{(2)}}(z)}{\mathrm{4(}n+\mathrm{1)}}{r}_{0}^{2}\\  &  & +\,\frac{(n+\mathrm{4)}{C}_{n,n}^{\mathrm{(4)}}(z)}{\mathrm{32(}n+\mathrm{1)(}n+\mathrm{2)}}{r}_{0}^{4}-\ldots ),\end{array}$$

for the unknown *C*_*n*,*n*_. This can be done by a Fourier transform of the measured ${\tilde{B}}_{n}({r}_{0},z)$, that is,

12 $$ {\mathcal F} \{{C}_{n,n}(z)\}=\frac{ {\mathcal F} \{{\tilde{B}}_{n}({r}_{0},z)\}}{ {\mathcal F} \{{k}_{n}({r}_{0},z)\}}\frac{-1}{{\mu }_{0}\,{r}_{0}^{n-1}(n-\frac{(n+\mathrm{2)(}i\omega {)}^{2}}{\mathrm{4(}n+\mathrm{1)}}{r}_{0}^{2}+\frac{(n+\mathrm{4)(}i\omega {)}^{4}}{\mathrm{32(}n+\mathrm{1)(}n+\mathrm{2)}}{r}_{0}^{4}-\ldots )}.$$

**Figure 1:**
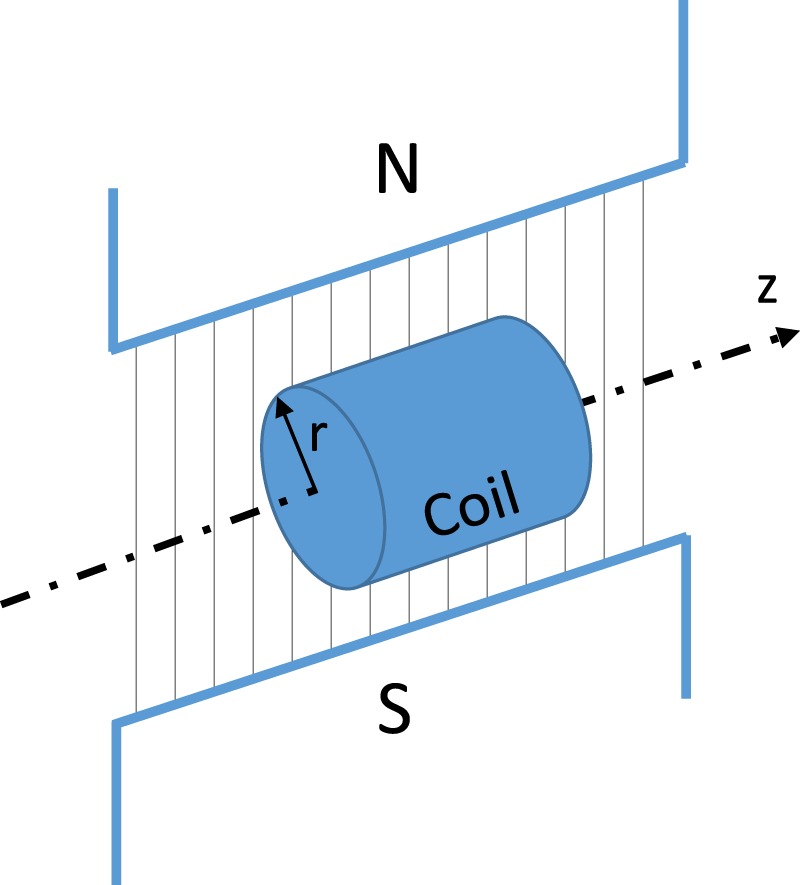
Magnets directions: (i) longitudinal, axis *z*; (ii) radial, radius *r*; and (iii) transversal, flux field lines.

In Fig. 1, the axis *z* represents the longitudinal direction of the magnet, the radius *r* is on the radial direction, and the transversal direction is represented by the flux lines.

### The Measurement Principle

The rotating-coil magnetometer is displaced step-by-step longitudinally along the magnet axis to measure the (convoluted) multipole-field errors as functions of the *z*-position. These functions are then deconvoluted by the kernels defined by the coil’s sensitivity functions of the corresponding harmonic order. The challenge is now to find a suitable order *n* of the pseudo-multipoles *C*_*n*,*n*_ and the highest-order derivatives ${C}_{n,n}^{(m)}$, in order to minimize the reconstruction uncertainty of the local magnetic field. The uncertainty of the method will also depend on the step size chosen for the longitudinal displacement of the transducer. Using computed field distributions and boundary values, a metric for the reconstruction uncertainty can be given by the residual *R*_*B*_ expressed as the normalized root-mean-square error:

13 $${R}_{B}=\frac{1}{{B}_{y}(K\mathrm{/2)}}\,\sqrt{\frac{\sum _{k=1}^{K}{[{B}_{y}(k)-{B}_{y}^{p}(k)]}^{2}}{K}},$$

where:

*B*_*y*_(*k*): the *y* component of the reference field distribution,

${B}_{y}^{p}(k)$: the reconstructed *y* component of the field distribution,

*k*: the index of the sampling point,

*K*: the maximum number of sampling points,

*B*_*y*_(*K*/2): the reference field component at the magnet center position.

**Figure 2:**
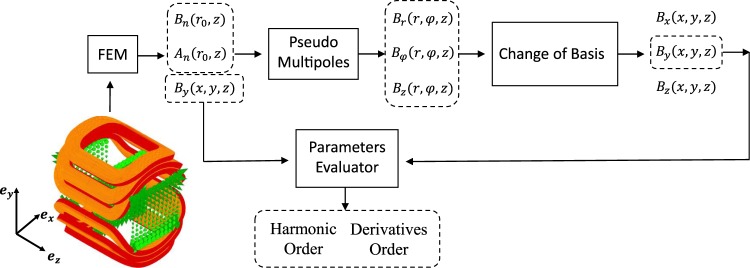
Method for assessing the design parameters: harmonic order *n* and derivative order *m*.

Figure 2 shows the procedure for finding the maximum orders for *n* and *m*. The longitudinal distributions of the normal and skew harmonics are computed by means of the CERN field computation program ROXIE^18^ for a short, air-coil corrector dipole magnet, as shown on the left of Fig. 2. The field harmonics were computed at a 50 mm reference radius, sampling every 1.2 mm along the magnet axis. The excitation current of the air-coil was set to 10 A, yielding a central field *B*_1_ of 37 mT. The optimum orders for *n* and *m* yield the functional specification for the induction-coil design. In particular, the maximum harmonic order imposes the coil opening angle, while the highest order derivative defines the required signal-to-noise ratio and imposes the sampling distance along the magnet axis.

**Figure 3:**
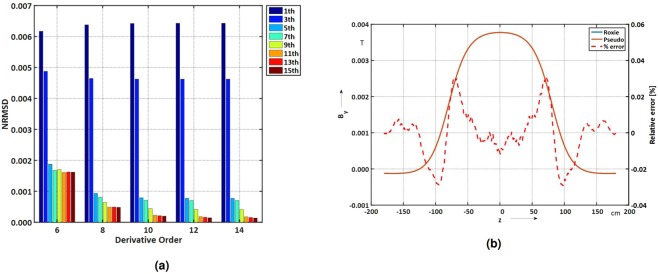
(**a**) Numerical results of the field reconstruction residual *R*_*B*_ versus derivative *m* and harmonic order *n*. (**b**) *B*_*y*_ field component and reconstruction error (in percent) along *z*. *n* = [1, 9] and *m* = [2, 10].

Results of this analysis are shown in Fig. 3a, where the residuals are assessed for different combinations of *n* and *m* for the reconstruction of the field along a line on the magnet’s vertical plane (at position *y* = 50, *x* = 0 mm, which is at about 2/3 of the magnet bore radius). The roll-off at the magnet extremity is relatively smooth for the low-order dipole and sextupole components, therefore considering the higher-order pseudo-multipoles for derivatives *m* > 10 yields no improvement. Figure 3a also shows that the multipoles up to *B*_9_ (i.e., *n* = 9) must be considered. For simulated field and boundary data, oversampling, (the maximum step size is established by taking the highest spatial frequency in the distribution of the *b*_3_ component (*m* = 12), which in the case of the air-coil dipole corresponds to 10 mm, and consequently sampling the domain with a step size of 5 mm), along *z* does not improve the result, but can be useful for (noisy) data acquired form the magnetic field transducer. Figure 3b shows the *B*_*y*_ field component and the reconstruction error (in percent) for the pseudo-multipole analysis with *n* = [1, 9] and *m* = [2, 10]. The highest error occurs in the fringe field region where the field distribution has the fastest roll off.

## Results

### Sensor Design

**Figure 4:**
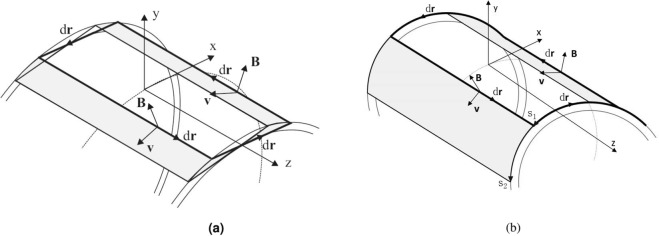
Sensor geometry: (**a**) tangential and (**b**) saddle-shaped, iso-perimetric coil.

The pseudo-multipoles are computed from the stepwise measurements of the transversal field components along the magnet axis. Therefore, the rotating-coil sensor must be insensitive to the longitudinal field component present in the magnet extremities. In other words, the integral over the **v** × **B** term along the coil end of the induction coil must be zero. When the induction coil is rotated, the integrated voltage is proportional to the flux intercepting the surface traced by this rotation, see Appendix A. This is shown in Fig. 4a for the classical tangential coil. Notice the surface elements that intercept the longitudinal field component. The proposed solution, shown in Fig. 4b, does not span any transversal surface when rotated about its axis.

**Figure 5:**
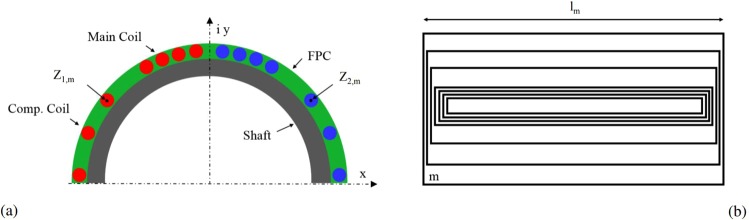
Cross-section of Flexible Printed Circuit (FPC) induction coil, with main and compensation coils (**a**) top view (**b**).

The objective is to design a sensor with enough sensitivity to the higher-order field harmonics and the possibility to compensate for the main field component that is by four orders of magnitude higher. An engineering solution is to nest several induction coils on a cylindrical shaft. Figure 5a shows the cross-section of the conceptual design. For an iso-perimetric coil, each turn remains on the same radius, and thus is not affected by the longitudinal field component when rotated around its longitudinal axis. Only the perpendicular field components will induce an electric field on the turn.

**Figure 6:**
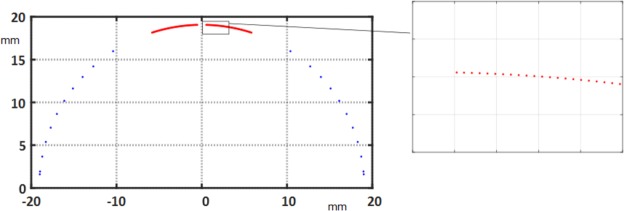
Optimized design of the dipole compensated coil with a magnified view of the inner coil (insert).

The sensor design is based on the equations for the complex sensitivity factors *K*_*n*_ of a single coil turn:

14 $${K}_{n}:\,={K}_{n}^{{\rm{rad}}}+i{K}_{n}^{\tan }=\frac{Nl}{n}({r}_{2}^{n}{e}^{in({\phi }_{2}-\phi )}-{r}_{1}^{n}{e}^{in({\phi }_{1}-\phi )}),$$

with their physical unit $[{K}_{n}^{{\rm{rad}}}]=[{K}_{n}^{\tan }]={{\rm{m}}}^{n+1}$, i.e., square meters for the dipole sensitivity. The superscript “rad” indicates the radial component and “tan” the tangential component, *r*_1_ and *r*_2_ are the radii of the go and return tracks, *n* is the multipole order, and *φ*_1_ and *φ*_2_ are the angular positions of the tracks. Thus, for *M* loops we obtain

15 $${K}_{n}=\sum _{m=1}^{M}\,\frac{N{l}_{m}}{n}({z}_{\mathrm{2,}m}^{n}-{z}_{\mathrm{1,}m}^{n}),$$

where *M* is the number of loops in the induction coils, *N* is the number of layers, *l*_*m*_ the length of the single loop of index *m*, *n* the harmonic order, and *z*_1,*m*_ and *z*_2,*m*_ are the complex coordinates of the *m*-th loop (see Fig. 5a and b). The design was optimized by means of the CERN field-computation program ROXIE. Two independent coils are combined on a common shaft. The central coil, with the smaller opening angle, is sensitive to higher-order field harmonics. The lower coil (with larger spacing between the turns) is designed to be sensitive only to the main dipole field component. From the theory of cos Θ coils^1^, we know that a single shell of *π*/3 rad creates the smallest amount of higher-order field components outside the shell and therefore minimizes the mutual inductance coefficient to the magnet. The two induction coils are then connected in series with opposite polarity so that the main field component is canceled out, and thus the signal-to-noise ratio is increased. Figure 6 shows the result of the mathematical optimization: In red, the position of the main induction coil and, in blue, the compensation coil.

**Figure 7:**
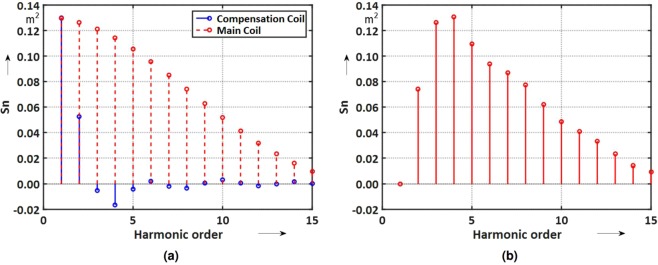
Sensitivity factors *S*_*n*_ at *R*_*ref*_ = 19.065 mm for the main and compensation coils (**a**) and for the compensated scheme (**b**).

The computed (design) sensitivity factors for the main and compensation coils are shown in Fig. 7a. The results for the compensation scheme are shown in Fig. 7b. As the higher-order sensitivity factors scale with 1/*r*^*n*−1^ it is appropriate to introduce a scaling factor, that is, the measurement reference radius *R*_ref_ and define

16 $${S}_{n}:\,=\frac{{K}_{n}}{{R}_{{\rm{ref}}}^{n-1}},$$

where *r* is the measurement radius.

**Figure 8:**
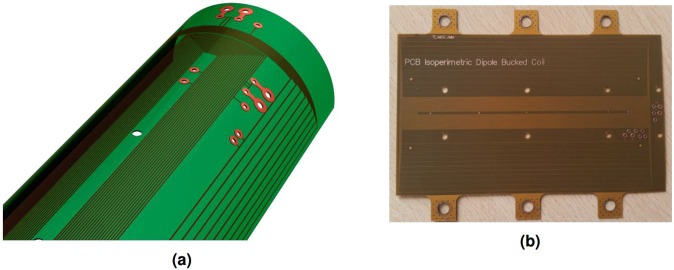
(**a**) 3D rendering of the coil design. (**b**) Photograph of the first printed circuit prototype.

An important feature is the absence of a “blind eye” for field harmonics up to the 15-th order. The blind eye is the multipole order *n* at which the opening angle (*δ*) is an integer fraction of 2*π*. In other words, the coil is completely insensitive to a multipole of order *n* when *nδ* = 2*π*. The ideal compensation of the main field component is a mathematical abstraction, however. Manufacturing tolerances make it impossible to produce nested coils spanning identical surfaces. As a quality factor, the compensation ratio (also known as bucking ratio) is defined as:

17 $${Q}_{{\rm{c}}}=\frac{{S}_{1}^{{\rm{m}}}}{{S}_{1}^{{\rm{m}}}-{S}_{1}^{{\rm{c}}}}$$

where ${S}_{1}^{{\rm{m}}}$ and ${S}_{1}^{{\rm{c}}}$ are the dipole sensitivities of the main and compensation coils. Compensation ratios on the order of 100 are usually considered as an achievement. Figure 8a shows a 3D rendering of the coil design, and Fig. 8b shows a photograph of the first prototype.

### Sensitivity Factors and Sensor Length

**Figure 9:**
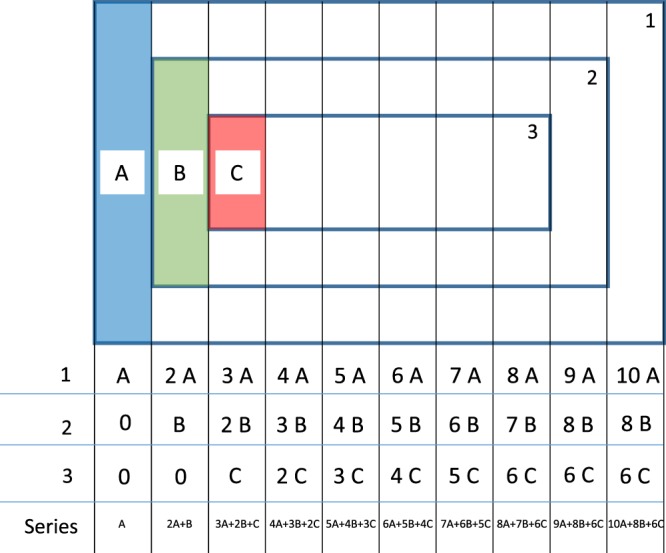
Schematic of the sensitivity factor analysis.

Rotating-coil magnetometers are usually designed to be longer than the magnetic length of the magnet under test, or at least long enough to cover the entire fringe field area. For this reason, the sensitivity factors are given for the geometric mean lengths of the magnetometer. For short coils, however, the coil-track thickness cannot be neglected with respect to their overall length. In particular, for coils produced in PCB (Printed Circuit Boards) technology, a certain gap size is required between the single turns, which increases the track thickness and limits the maximum number of turns. Therefore, the sensitivity factors must be expressed locally as a function of the longitudinal position within the coil. This yields the kernel for deconvoluting the measured multipole distribution. Let us consider a one-layer, flexible printed circuit of three induction coil tracks with external length *L* of 10 cm and a spacing of 1 cm between them, as illustrated in Fig. 9.

The spanned surface (i.e. the *K*_1_ value) for a series connection of the three turns is given by the cumulative sum over the contributions from the three turns, that is, the last row in Fig. 9. The finite difference of this sum yields the sensitivity function (convolution kernel) *k*_1_(*z*). Considering a flexible printed circuit with *M* turns, a total length *L*, and a step size *t* given by the distance between each turn in the coil end, we get for the *n*-th harmonic,

18 $${V}_{n}(m,i)=\frac{t(i-m+1)}{n}({z}_{2,m}^{n}-{z}_{1,m}^{n}),\,{\rm{for}}\,\,i > m,$$

where *m* ∈ 1, 2, …, *M* and *i* ∈ 1, 2, …, *I*, *I* = *L*/*t*. The sums of the contributions at *I* (last column in Fig. 9) are the sensitivity factors corresponding to the ones shown in Fig. 7a.

19 $${K}_{n}=\sum _{m=1}^{M}\,{V}_{n}(m,I).$$

**Figure 10:**
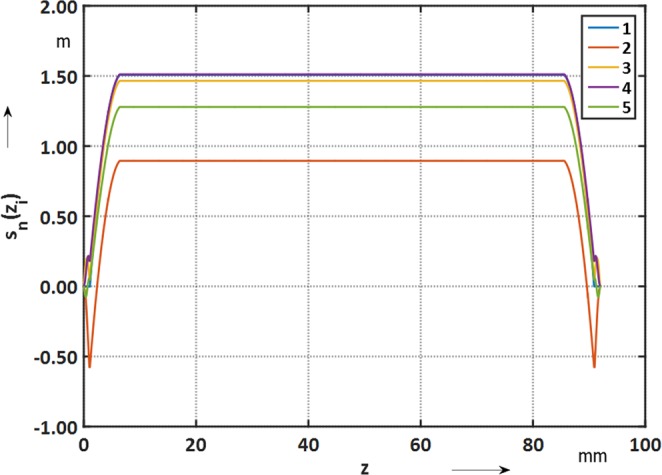
Compensated coil sensitivity along the induction coil (*R*_*ref*_ = 19.065 mm).

In order to calculate the sensitivity function *k*_*n*_(*z*_*i*_), the finite difference of the sums over *V*_*n*_(*m*, *i*) is required:

20 $${k}_{n}({z}_{i})=\frac{\sum _{m=1}^{M}\,{V}_{n}(m,i)-\sum _{m=1}^{M}\,{V}_{n}(m,i-1)}{t},$$

for *i* ∈ 2, 3, …, *I*. The functions ${s}_{n}({z}_{i})={k}_{n}({z}_{i})/{R}_{ref}^{n-1}$ for the induction coil are given in Fig. 10.

**Figure 11:**
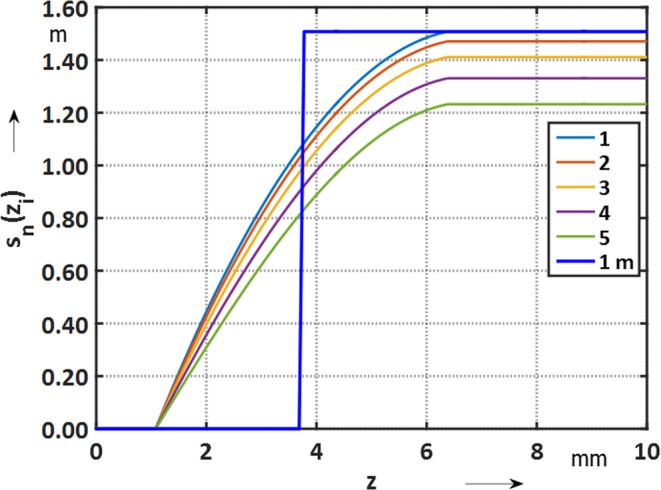
Sensitivity functions *s*_*n*_(*z*_*i*_) along the extremities of the main induction coil and hard-edge model (geometric mean length).

The main difference between the classical approach (coils that have a track thickness that is negligible with respect to the coil length), and the printed circuit technology, is that its sensitivity function varies with the multipole order; see Fig. 11. Therefore, the geometric mean length does not correspond to the magnetic length. The deviation from the “hard-edge model”, which is employed in case of the classical coils is shown in Fig. 11.

**Figure 12:**
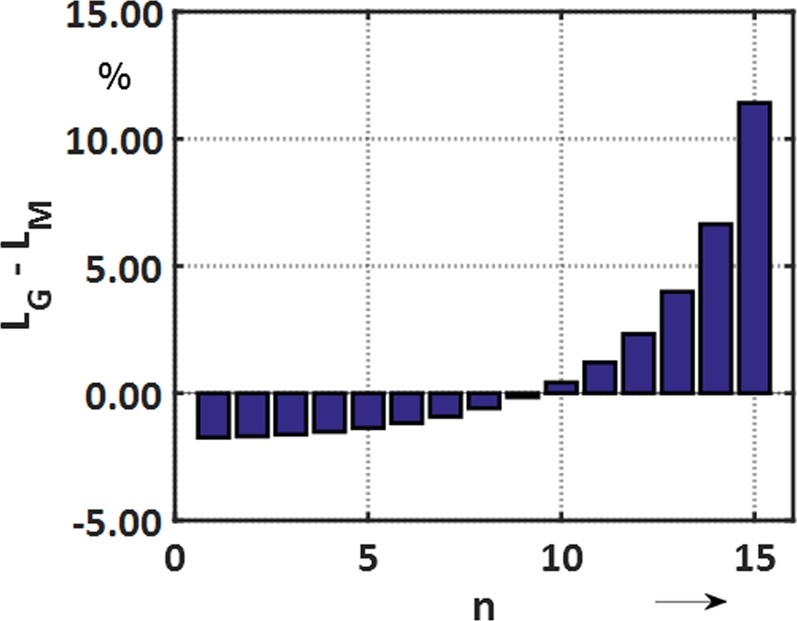
Differences between the geometric mean and magnetic lengths as a function of the multipole order n.

The differences between the geometric mean and magnetic lengths (*L*_*G*_ and *L*_*M*_) are given in Fig. 12 for the multipole order n.

### Uncertainty Analysis

The sensor performance, in terms of compensation ratio and sensitivity, is affected by manufacturing tolerances during the PCB production. The uncertainty on the sensitivity factors is analyzed in order to derive the required production tolerances. Both random and systematic errors on the track positioning are considered. Uniformly-distributed, pseudo-random errors in the range of ±30 μm are considered for the track positions. The complex coordinates of each track are

21 $${z}_{m}=r\,\cos \,(\frac{a+x}{r})+i\,r\,\sin \,(\frac{a+x}{r}),$$

where *a* is the nominal arc length calculated by ROXIE, *r* is the nominal radius of the shaft, and *x* the random error. The assumption of a random error not exceeding ±30 μm is reasonable, because larger errors would lead to short circuits because the insulating thickness between turns is only 50 μm. The most sensitive parameter for quantifying the track-positioning error is the compensation ratio, see Eq. 17. Table 1 shows the resulting compensation ratios for different levels of random errors.

**Table 1:** Compensation ratios for different track-positioning errors.

Position uncertainty	Dipole compensation ratio
none	130000
±20 μm	17000
±30 μm	16000

**Figure 13:**
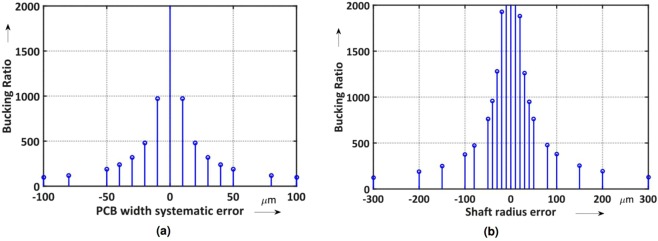
Compensation ratio of a dipole-compensated coil with systematic error in PCB width (**a**) and compensation ratio of a dipole-compensated coil at varying the tolerance on the shaft radius (**b**).

The flexibility of the induction coil sensor may result in a lengthening or compression of the printed circuit during assembly on the shaft and therefore may result in systematic errors on the track positions. Simulations were carried out considering a maximum error of ±100 μm on the total width. Figure 13a shows the results of the compensation ratio for a dipole-compensated coil. The maximum acceptable systematic error that will ensure a compensation ratio higher than 100 is therefore ±80 μm. Apart from mounting/gluing the flexible printed circuit on the shaft, further significant errors arise from the radius tolerance on the shaft itself. Simulations were carried out considering a maximum error of ±300 μm on the nominal radius of 19.065 mm. For a compensation ratio larger than 100, the shaft tolerance must be better than ±200 μm; see Fig. 13b.

### Sensor Production

The sensor was produced at CERN by the PCB service of the EP-DT-EF section. The first prototype is shown in Fig. 8b. The total length is 98.2 mm, the width is 61 mm and the thickness 240 μm. The main coil consists of 59 loops with an area of 0.129 m^2^ and magnetic length for the sensitivity *K*_1_ of 84.308 mm. The compensation coil consists of 11 turns with a magnetic length of the *K*_1_ sensitivity of 90.982 mm.

### Sensor Validation Experiments

**Figure 14:**
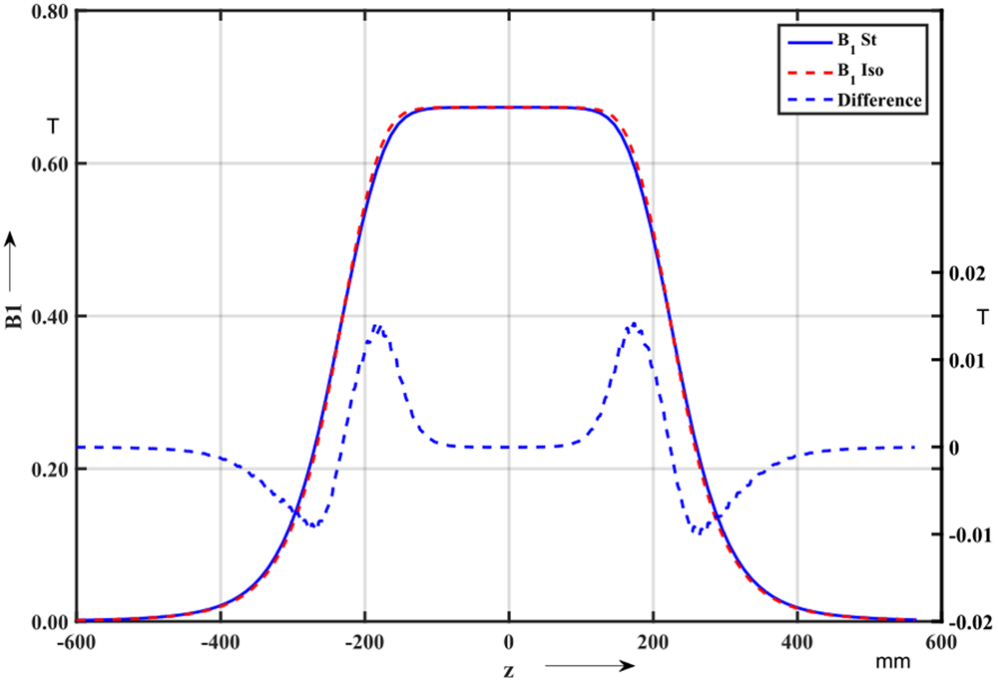
Main field profile along the magnet center axis measured with the iso-perimetric coil (dashed red) and the radial coil (solid). The dashed blue line is the difference in tesla.

As a first functional test of the prototype transducer, the electrical resistance was measured to verify the continuity between the four layers of the printed circuit. As a proof of principle, magnetic measurements were carried out on transfer-line dipole magnet, powered at 150 A. The dipole has an air gap of 100 mm × 134 mm. The longitudinal profiles of the field harmonics were measured both by a classical radial coil and by the new iso-perimetric coil. A first multipole scanning was performed along the center of the magnet, where the transversal field distribution is almost symmetric. Along this trajectory, the magnetic flux density has only *B*_3_, *B*_5_, and higher-order odd multipole components. In a second run, the magnet was mapped along a track located close to the pole (displaced track). In this way, higher-order skew and normal field components can be expected^19^. The axis of this scanning track is displaced by x = 20 mm and y = −30 mm from the center of the magnet bore. Figure 14 shows the main field profile at the magnet center. The dashed red and the continuous blue lines represent the profile measured with the iso-perimetric sensor and the classical radial coil, respectively. The dashed blue line represents the difference in tesla.

**Figure 15:**
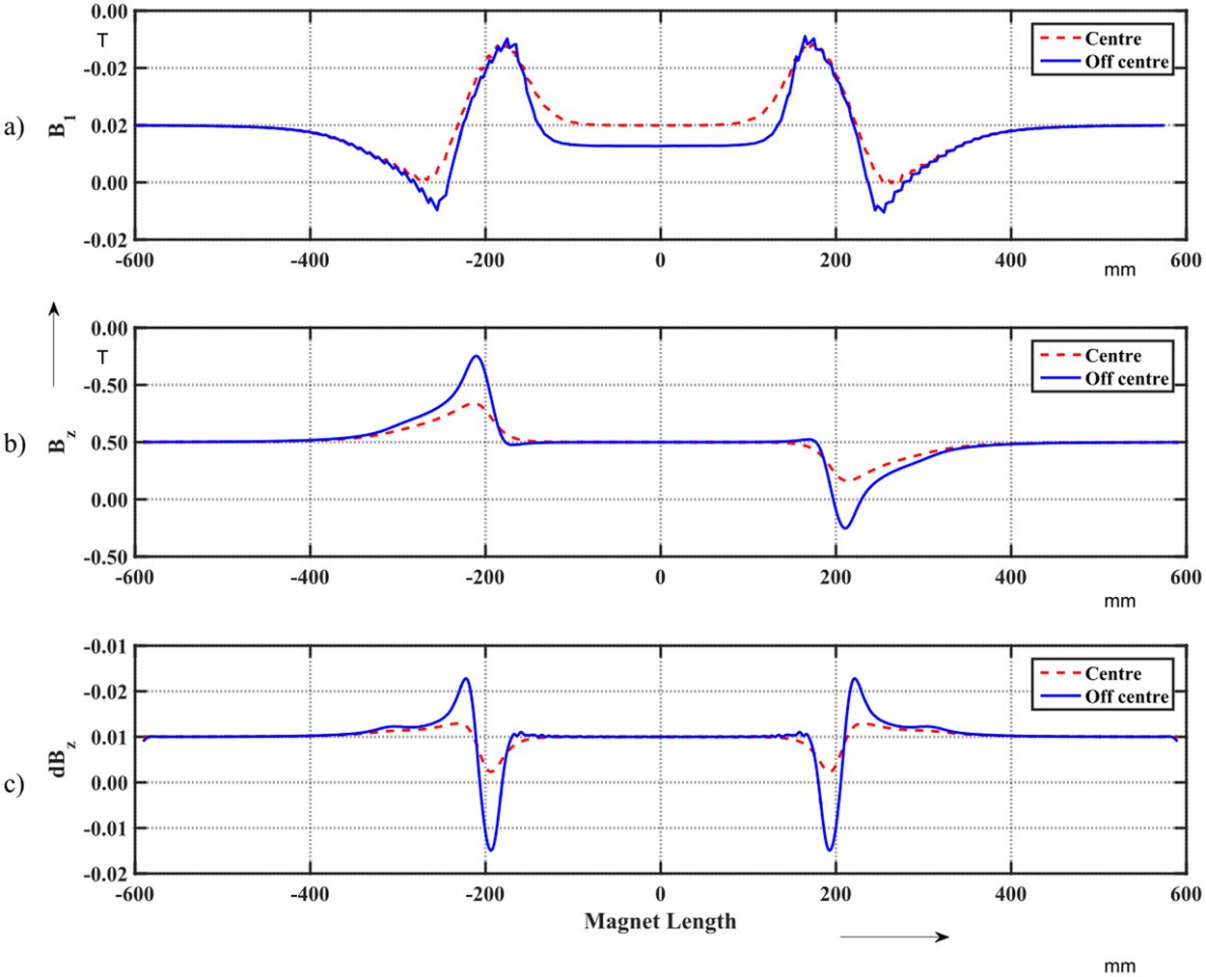
(**a**) Differences between the *B*_1_ field components measured with the classical and iso-perimetric coils. Red: For the scan along the central axis. Blue: For the scan along the displaced track. (**b**) Computed *B*_*z*_ field component as a function of the longitudinal position. Red: Central axis. Blue: Displaced track. (**c**) Derivative of the computed *B*_*z*_ component. Red: Central axis. Blue: Displaced track.

Figure 15a shows the differences between the *B*_1_ field components measured with the classical and iso-perimetric coils: the difference of the scan along the central axis (as in Fig. 14) is highlighted in red, and along the displaced track, in blue. Figure 15b shows the computed field component *B*_*z*_ as a function of the longitudinal position, and Fig. 15c the derivative of the computed component *B*_*z*_. The difference between the two measurements (using the classical and iso-perimetric coils) is higher in case of the displaced track. This is due to the quadrupole field component present along the displaced trajectory. In the magnet end regions, this component gives rise to higher asymmetric, longitudinal field components as seen in the in the numerical field computation^20^ (Fig. 15b). Using the classical coil, voltages are induced in the extremity, which affect the measured *B*_1_ component that, consequently, cannot be used for extracting pseudo-multipoles.

## Conclusions

A rotating-coil magnetometer was developed and realized at CERN. The aim is to measure the longitudinal magnetic field profiles of magnets with large fringe fields. The proposed rotating coil magnetometer is based on a flexible printed circuit mounted on a cylindrical shaft, such that all turns remain on the same radius (iso-perimetry). The uncertainty analysis yields production tolerances of ±80 μm (systematic) on track positions and ±200 μm on the shaft radius. The sensitivity factors for short PCB coils become functions of the coil length that varies with the multipole order. This sensitivity function is the kernel of convolution with the field distribution in the magnet. First results have demonstrated that the iso-perimetric coil is less affected by the longitudinal field components (compared to the classical design). This effect is more obvious when the magnet is measured along a trajectory displaced from the magnetic axis. The reason is the asymmetric field distribution in the magnet extremities.

## Supplementary information

Supplementary information
