# Supplementary material for: A Rotating-Coil Magnetometer for Scanning Transversal Field Harmonics in Accelerator Magnets
Source: Sci Rep. 2019 Feb 6;9:1491. doi: 10.1038/s41598-018-37371-3 (PMC6365526; doi:10.1038/s41598-018-37371-3)
Supplement: Supplementary file 1 — Supplementary information [file 41598_2018_37371_MOESM1_ESM.pdf]

A Rotating-Coil Magnetometer for Scanning Transversal Field Harmonics in Accelerator Magnets

Pasquale Arpaia[1], Gianni Caiafa[1,2,*], and Stephan Russenschuck[2]

[1]Instrumentation and Measurement for Particle Accelerators Laboratory, Department of Electrical Engineering and Information Technology, University of Napoli Federico II, Naples, Italy
[2]Magnetic Measurements Section, Technology Department, European Organization for Nuclear Research, Geneva, Switzerland
[*]gianni.caiafa@cern.ch

ABSTRACT

This paper presents a rotating-coil magnetometer that was designed and validated for scanning local transversal field harmonics, required for extracting so-called pseudo-multipoles in accelerator magnets. The magnetometer consists of four layers of flexible printed circuits with a track thickness of 40 μm. The design aimed at maximizing the sensitivity factors for field harmonics up to order 13 and at a compensation ratio for the main component in the same range of what is achievable with standard rotating coils. Key innovative features of the induction coil are the shape for minimizing the sensitivity to the longitudinal field component and the manufacturing technology. The design, the uncertainty analysis of the manufacturing tolerances, as well as preliminary application results are presented.

A. Appendix: Integration of the voltage induced in a rotating-coil magnetometer

The voltage acquired during the displacement of the magnetometer is integrated by means of a digital integrator at time $t_i(s_i)$. This integrator is triggered by the readout of an angular encoder. In this way the measurement is re-parametrized with respect to the arc length s, and becomes independent of the motion uniformity of the drive system.

$$\begin{aligned}
\int_{t_1(s_1)}^{t_2(s_2)} U(\partial\mathscr{A})\cdot \mathrm{d}t &= \int_{t_1(s_1)}^{t_2(s_2)}\int_{\partial\mathscr{A}} (\mathbf{v}\times\mathbf{B})\cdot \mathrm{d}\mathbf{r}\,\mathrm{d}t \\
&= \int_{t_1(s_1)}^{t_2(s_2)}\int_{\partial\mathscr{A}} -\mathbf{B}\cdot(\mathbf{v}\times \mathrm{d}\mathbf{r})\mathrm{d}t \\
&= \int_{t_1(s_1)}^{t_2(s_2)}\int_{\partial\mathscr{A}} -\mathbf{B}\cdot(\mathbf{v}\mathrm{d}t)\times \mathrm{d}\mathbf{r} \\
&= \int_{\partial\mathscr{A}}\int_{s_1}^{s_2} -\mathbf{B}\cdot(\mathrm{d}\mathbf{s}\times \mathrm{d}\mathbf{r}) \\
&= \int_{\mathscr{A}_\mathrm{s}} -\mathbf{B}\cdot \mathrm{d}\mathbf{a}\,,
\end{aligned} \tag{1}$$

where $\mathscr{A}_\mathrm{s}$ is not the surface spanned by the induction coil, but the surface traced out by the rim of that surface during the displacement between two angular positions (arc lengths s_1 and s_2); see the gray surface in the sensor geometry and Fig. 1.

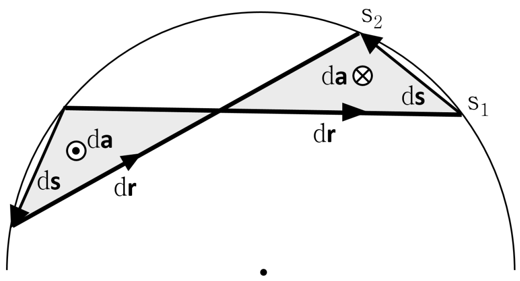

Figure 1. Cross-sectional view of a radial coil turn displaced between two angular positions of arc lengths s_1 and s_2.
